# Atlas of Cutaneous Diseases

**Published:** 1856-08

**Authors:** 


*Atlas of Cutaneous Diseases.* By J. MOORE NELIGAN, M. D., Edin., M. R. J. A., etc., etc. Philadelphia: BLANCHARD & LEA.

This beautiful atlas comes to us unaccompanied by the treatise which forms its text. The two together must constitute a very valuable work. The plates of the atlas are in the very best style of lithographic art, and are remarkable for truthfulness of coloring. The entire number of plates is sixteen; each divided into six compartments; thus illustrating some ninety-six different forms of cutaneous disease. Each is accompanied by an explanatory paragraph, written in a close, condensed style. The work will form a very valuable assistant to the student of cutaneous diseases. It has no equal in point of typographical excellence.

